# Air versus sulfur hexafluoride gas tamponade for small- and medium-sized macular holes in prone and non prone positions: a randomized controlled study

**DOI:** 10.1186/s40942-026-00806-5

**Published:** 2026-02-12

**Authors:** Pallavi Goel, Tanya Jain, Manisha Agarwal, Shalini Singh, Divyansh Kailashchandra Mishra

**Affiliations:** 1 Department of Vitreo Retina, Dr. Shroff’s Charity Eye Hospital, Delhi, India; 2 Department of Vitreo Retina, Sankara Eye Hospital, Bengaluru, India

**Keywords:** Macular hole, Air, SF6, Prone positioning

## Abstract

**Purpose:**

To compare the anatomical and functional outcomes of patients with small- to medium-sized idiopathic macular holes (MHs) treated with air or sulfur hexafluoride (SF6) gas tamponade in prone and nonprone positions.

**Methods:**

This was a multicentric randomized study of patients who were diagnosed with idiopathic small- to medium-sized (minimum linear diameter 250–400 μm) full-thickness macular holes and who underwent pars plana vitrectomy with inverted internal limiting membrane peeling. Patients were randomized into three groups: Group 1, air tamponade with prone positioning; Group 2, SF6 with prone positioning; and Group 3, SF6 without prone positioning. The primary outcomes assessed were the anatomical closure of MH and the type of MH closure. The secondary outcomes assessed were changes in best-corrected visual acuity (BCVA), postoperative changes on OCT and intraocular pressure (IOP).

**Results:**

Eighty patients were enrolled in the study. The overall degree of anatomical closure was 92.5% (74/80). 88% (87.88% i.e. 21/22) in group-1, 100% (33/33) in Group-2 and 80% (20/25) in Group-3. The percentages of persistent MHs were 4.55% (1/22), 0% (0/33) and 20% (5/25) in groups 1, 2 and 3, respectively. The BCVA of patients improved from LogMAR 0.69 ± 0.29 to LogMAR 0.41 ± 0.29 at one month. BCVA improved across all groups postoperatively, with no significant differences at 1 month (*p* = 0.68) between all three groups. The photoreceptor (PR) layer defect length was greatest in the SF6 + non prone group (536.88 ± 341.1 microns), followed by the air + prone group (447.47 ± 346.35 microns) and least in the SF6 + prone group (272.5 ± 101.82 microns) at postoperative day 10. The PR layer defect was significantly lower in the SF6 + prone group and the air + prone group than in the SF6 + nonprone group (*p* = 0.049). IOP elevation occurred more frequently in the SF6 groups than in the air group (*p* = 0.015).

**Conclusion:**

Air tamponade is a viable alternative to SF6 for small to medium MHs in the prone position. It achieved comparable anatomical and functional outcomes while reducing IOP-related complications. Nonprone positioning, although convenient, results in a greater incidence of persistent holes, emphasizing the critical role of postoperative positioning even in small- and medium-sized MHs.

**Trial registered:**

The trial was registered with the clinical trial registry of India (CTRI/2023/05/067593);dated 19/05/2023

## Background

Macular holes (MHs) are full-thickness defects in the central retina that often lead to significant visual impairment if left untreated [1]. These defects are most commonly idiopathic but can also result from trauma, high myopia, or other retinal conditions. Macular hole is a relatively common indication for vitreoretinal surgery, particularly in older patients. The standard surgical approach includes pars plana vitrectomy (PPV), peeling of the internal limiting membrane (ILM), and the use of intraocular tamponade to facilitate retinal apposition and promote hole closure [2]. Tamponade helps in MH closure by preventing fluid flow into the hole, reducing retinal edema, and creating interfacial surface tension to pull retinal edges together [2]. 

A variety of tamponading agents have been used, including air, sulfur hexafluoride (SF6), hexafluoroethane (C2F6), perfluoropropane (C3F8), and silicone oil. Among these, SF6 gas has long been favoured because of its longer retention time and proven efficacy compared with those of air [3]. However, there is growing interest in air as an alternative for small and medium–sized MHs, offering shorter absorption times, reduced patient discomfort, decreased IOP alterations and inflammation with earlier visual recovery.

Postoperative positioning is another critical consideration, as prone positioning enhances tamponade effectiveness but is often inconvenient and poorly tolerated. Emerging evidence suggests that nonprone positioning may be effective in the closure of MHs, further improving patient compliance and satisfaction.

This study aimed to compare the efficacy of air versus SF6 gas tamponade in the closure of small- to medium-sized macular holes, assessing anatomical and functional outcomes with prone and nonprone positioning. We also evaluated patient comfort and tolerability to prone positioning for optimization of the surgical results.

### Methodology

A prospective, randomized, multicentric interventional study was conducted at two tertiary eye hospitals located in North India and South India. Consecutive patients with small- to medium-sized macular holes were enrolled in the study between 1st December 2022 and 31st August 2024.

Primary and secondary MHs were defined according to the international vitreomacular traction study group classification [4]. 

The inclusion criteria for the study were patients > 18 years of age, duration of symptoms < 6 months, primary idiopathic macular holes, small- to medium-sized macular holes with a maximum base diameter of ≤ 400 microns, [4] and the ability of patients to provide informed consent.

The exclusion criteria were patients < 18 years of age, secondary MHs, high myopia (Axl > 26 mm), previous vitreoretinal surgery or any ocular comorbidity.

Institutional ethics committee approval was obtained at all sites, and the study followed the Declaration of Helsinki. The trial was registered with the clinical trial registry of India (CTRI/2023/05/067593).

Patient follow-up was performed at postoperative days 1, 10, 30 and 6 week.

At each follow-up, a complete ophthalmic examination was performed, including best corrected visual acuity (BCVA) via Snellen’s chart, which was converted into LogMAR for statistical analysis and anterior and posterior segment examination. Intraocular pressure (IOP) was measured via Goldmann applanation tonometry. Optical coherence tomography (OCT) was conducted via Zeiss spectral domain OCT.

The size of the macular hole (MH) was determined according to the International Vitreomacular Traction Study Group classification [4]. Parameters such as base diameter, minimum linear diameter (i.e., the narrowest point in the mid-retina parallel to the retinal pigment epithelium [RPE]), and photoreceptor layer defects were recorded via spectral-domain OCT (SD-OCT).

Patients scheduled for surgery were randomized into one of three groups prior to surgery via envelope method/chit system of simple randomization.The patients were randomly allocated to one of the three groups after providing informed consent.

Group 1: Air + prone positioning.

Group 2: SF6 (20%) + prone positioning.

Group 3: SF6 (20%) + non prone positioning.

The surgery was performed by 3 surgeons who have been practicing vitreoretinal surgery for more than 10 years, using a standard 3-port, 25-gauge pars plana vitrectomy to release anterior, posterior and tangential traction. The internal limiting membrane (ILM) was peeled using Brilliant Blue G (BBG) dye assistance and the inverted ILM flap technique with Grieshaber Revolution ILM Forceps. The size of ILM peel (In optic disc diameter) was documented by the operating surgeon via intraoperative observation. After fluid‒air exchange, the eye was either left with air tamponade or SF6 (20%). The 20% SF6 was prepared by drawing 10 ml gas in 50 ml syringe from the gas cylinder using microfilter and then reconstituted with 40 ml air. Patients with any intraoperative complication requiring a change in the protocol of the surgery were excluded from the study. Patients in group requiring prone position, were asked to maintain the face down position for atleast 8 h a day for 5 days postoperative.

Each patient was followed up on the 1st, 10th, and 30th days and at 6 weeks after surgery. OCT was evaluated at day 10, 30, and 6 weeks postoperative by a blinded examiner unaware of the tamponade used. In patients whose MH persisted by the 10th day in either group, an additional SF6 gas injection was administered.

Type-I closure is defined as the closure of the MH with no interruption in the continuity of foveal tissue above the retinal pigment epithelial layer [5]. 

Type II closure of the MH involves interruption of the continuity of the foveal tissue. The hole edges are thinned and attached to the underlying retinal pigment epithelial layer [5]. 

Surgical success was defined as a type I or type II closure of the MH.

The primary outcomes assessed were the degree of MH closure and the type of MH closure. The secondary outcomes assessed were changes in best-corrected visual acuity (BCVA), postoperative changes on OCT and intraocular pressure (IOP) .

### Statistical analysis

The categorical variables are presented as numbers and percentages (%). The quantitative data are presented as the means ± SDs and as medians with 25th and 75th percentiles (interquartile ranges). The normality of the data was checked via the Shapiro‒Wilk test. For the cases in which the data were not normal, we used nonparametric tests. The following statistical tests were applied for the results:

The variables that were quantitative and not normally distributed in nature were compared via the Mann‒Whitney test (for two groups) and the Kruskal‒Wallis test (for more than two groups), and the variables that were quantitative and normally distributed in nature were analysed via the independent t test (for two groups) and ANOVA (for more than two groups). A post hoc test (Bonferroni correction) was applied after ANOVA. For all non normally distributed data, post hoc analysis with Dunn’s multiple pairwise comparison test was carried out.

The variables that were qualitative in nature were compared via the chi-square test. If any cell had an expected value of less than 5, Fisher’s exact test was used.

The Pearson correlation coefficient was used to assess the correlation of BCVA at 1 month with base and mid diameters. The point biserial correlation coefficient was used to correlate the ILM (DD) with the DONFL.

The data were entered into the Microsoft EXCEL spreadsheet, and the final analysis was performed via the Statistical Package for Social Sciences (SPSS) software, IBM manufacturer, Chicago, USA, version 25.0.

A p value of less than 0.05 was considered statistically significant.

## Results

A total of 80 eyes from 80 patients with a male-to-female ratio of 0.63:1 (49 females and 31 males) and idiopathic macular holes were included in the study. The mean age of the patients was 63.6 ± 11.78 years.

### Anatomical outcomes

#### Type of closure

**Fig. 1:**
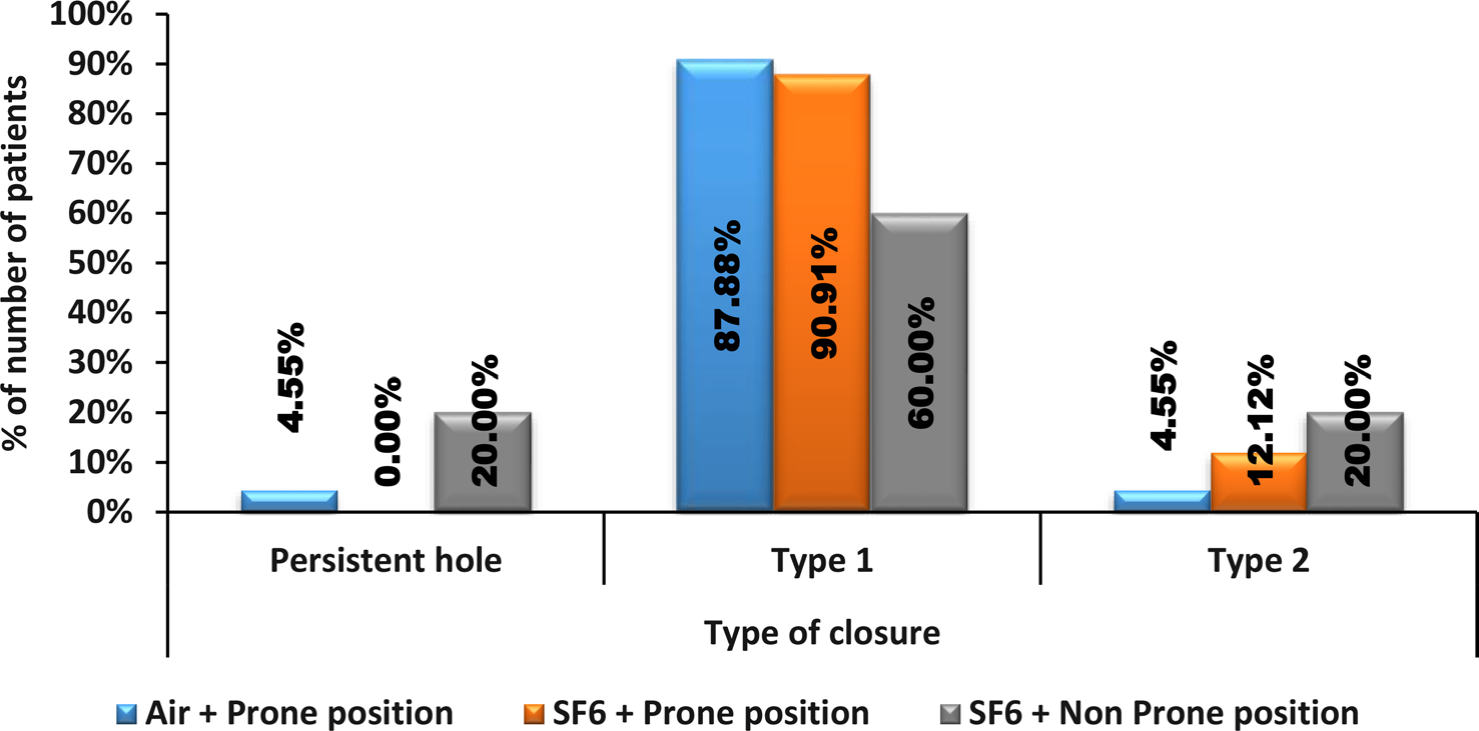
Comparison of type of closure between Air + Prone position, SF6 + Prone position and SF6 + Non Prone position

The distribution of closure types revealed significant differences between all three groups (p value = 0.015). The proportion of persistent holes was significantly greater in the SF6 + Non-Prone position group (20%) than in the Air + Prone position group (4.55%) and the SF6 + Prone position group (0%). For Type 1 closures, the proportion was highest in the SF6 + prone position group (90.91%), followed by the air + prone (87.88%) and SF6 + nonprone position (60%) groups. For Type 2 closure, the proportion was highest in the SF6 + Non-Prone position (20%), followed by the SF6 + Prone position (12.12%) and the Air + Prone position (4.55%). ( Fig. 1)

Pairwise comparisons revealed significant differences in persistent holes between the SF6 + prone position and the SF6 + non‒prone position (p value = 0.012). Comparisons between the Air + Prone position and the SF6 + Prone position (p value = 0.343) and between the Air + Prone position and the SF6 + Non-Prone position (p value = 0.056) were not statistically significant (Fig. 1).

**Fig. 2:**
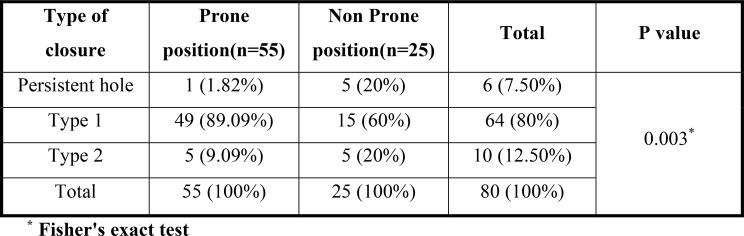
Type of macular hole closure in prone and non prone position

When compared with the nonprone position, the prone position had a significantly greater proportion of patients with Type 1 closure (89.09% vs. 60% in the prone and nonprone positions, respectively). Conversely, persistent hole (1.82% in prone vs. 20% in non prone) and Type 2 closure (9.09% in prone vs. 20% in nonprone) were significantly lower in the prone position (p value = 0.003) (Fig. 2).

### Ellipsoid zone layer disruption or photoreceptor (PR) layer defects

**Fig. 3:**
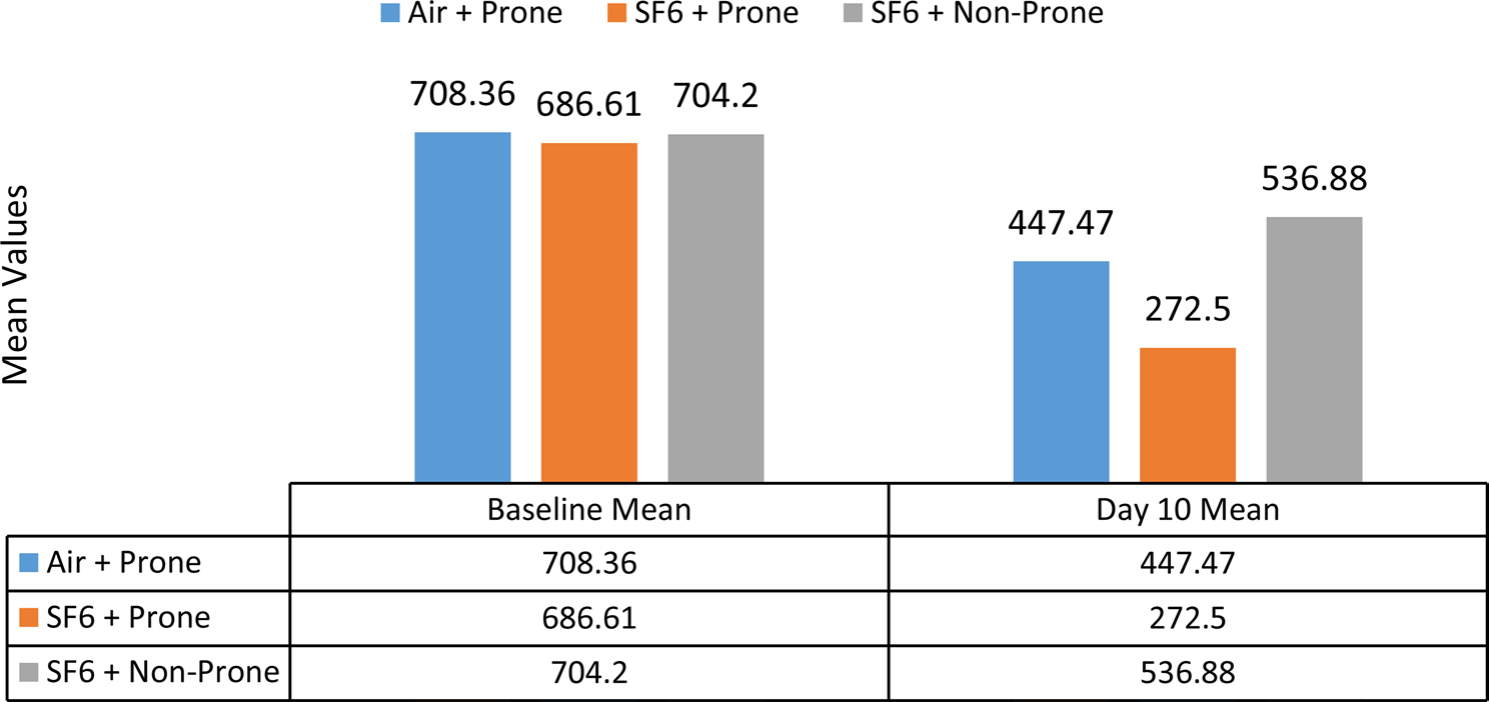
Comparison of length of PR layer defect between Air + Prone, SF6 + Prone and SF6 + Non Prone positions

The mean length of the PR layer defect on the 10th day was significantly different across the three groups i.e. air + prone, SF6 + prone and SF6 + non prone (p value = 0.034). The mean ± SD length was greatest in the SF6 + non prone group (536.88 ± 341.1 microns), followed by the Air + Prone position group (447.47 ± 346.35 microns), and it was lowest in the SF6 + Prone position group (272.5 ± 101.82 microns) (Fig. 3).

#### Effect of tamponade on PR layer defects

However, no significant difference was detected in the length of the PR layer defect at baseline or on the 10th day between different tamponades, i.e., air and SF6. Mean ± SD length of the PR layer defect at baseline: 823.36 ± 228.77 microns in air vs. 704.97 ± 260.92 microns in SF6 (p value = 0.065) and on the 10th day: 536.88 ± 341.1 microns in air vs. 347.49 ± 250.81 microns in SF6 (p value = 0.079).

#### Effect of positioning on the PR layer defect

A strong significant difference in the PR layer defect was noted on the 10th day between the prone and nonprone positions. The mean ± SD length of the PR layer defects at baseline was 741.31 ± 250.56 microns vs. 729.2 ± 274.67 microns (p value = 0.846), and on the 10th day, it was 348.04 ± 228.59 microns vs. 647.47 ± 346.35 microns (p value: 0.000376) in prone and non prone positions respectively.

### Size of the ILM and its association with the dissociated nerve fibre layer (DONFL)

**Fig. 4:**
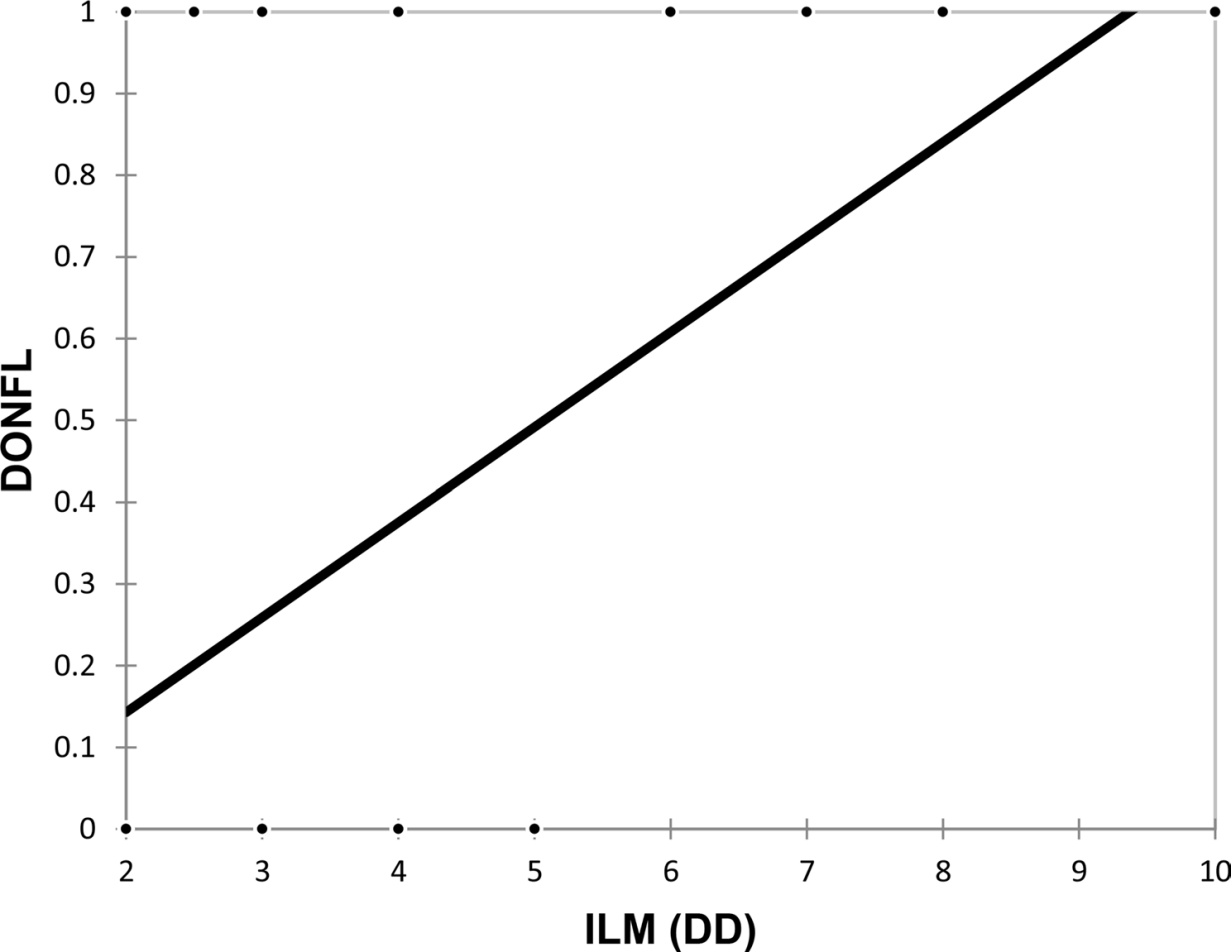
Correlation of ILM (DD) with DONFL

A significant moderately positive correlation was observed between the ILM (DD) and the DONFL, with a correlation coefficient of 0.49, indicating that a larger ILM peeled during surgery was associated with a greater likelihood of the formation of the DONFL (Fig. 4).

### Functional outcomes

#### Best corrected visual acuity (BCVA)

The mean BCVA at baseline was 0.82 ± 0.37 LogMAR in the SF6 + prone group, 0.61 ± 0.12 LogMAR in the air + prone group and 0.59 ± 0.24 LogMAR in the SF6 + non prone group. The mean ± SD change in BCVA was 0.37 ± 0.21 LogMAR in the air + prone position group vs. 0.44 ± 0.32 LogMAR in the SF6 + prone position group vs. 0.4 ± 0.31 LogMAR in the SF6 + non prone position group at 1.5 months.

**Fig. 5:**
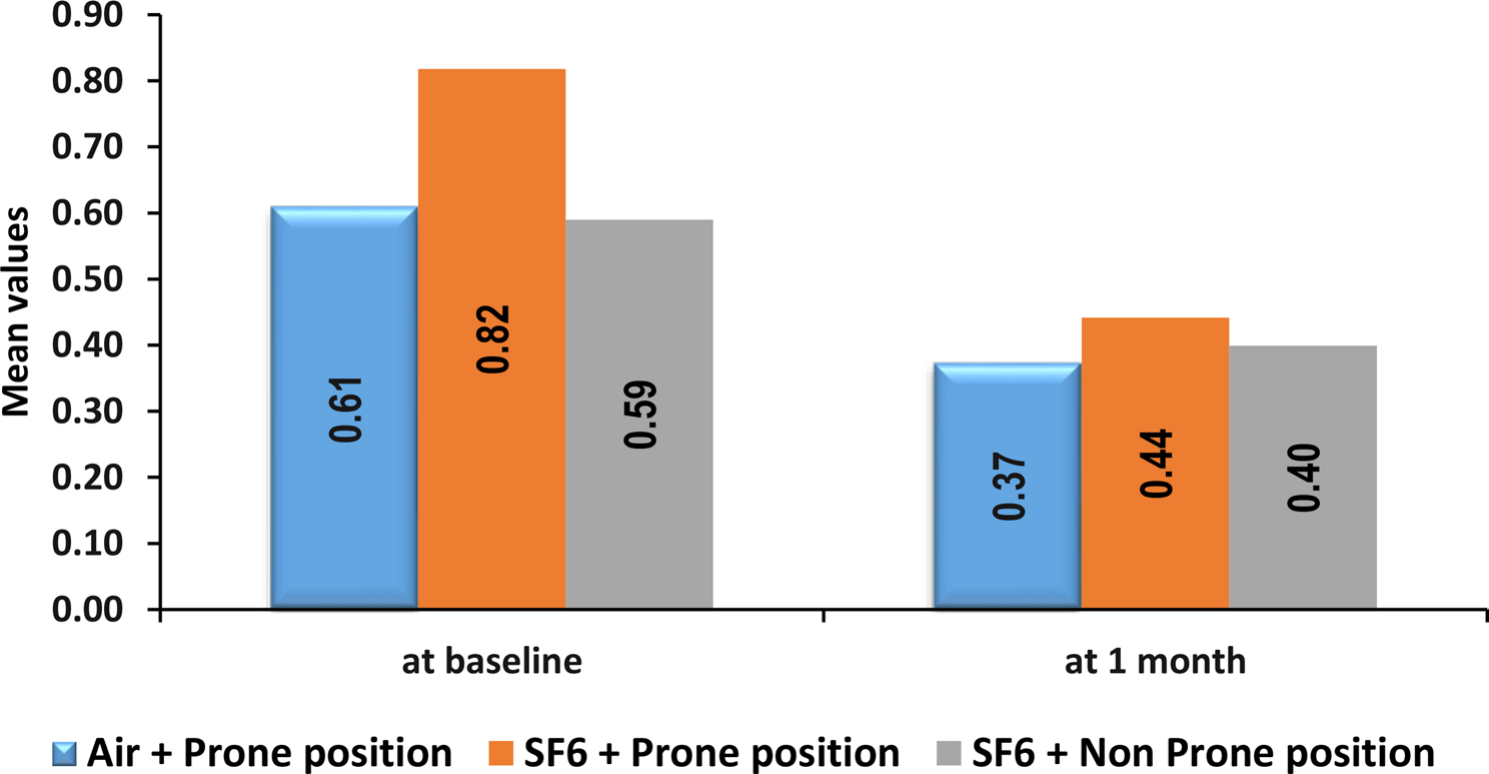
Comparison of BCVA (LogMAR) between Air + Prone position, SF6 + Prone position and SF6 + Non Prone position

The mean BCVA (logMAR) at 1.5 months (6 weeks) was not significantly different across all three groups (p value = 0.68). Pairwise comparisons revealed no significant differences between the Air + Prone position and the SF6 + Prone position (p value = 0.67), between the Air + Prone position and the SF6 + Non-Prone position (p value = 0.949) or between the SF6 + Prone position and the SF6 + Non-Prone position (p value = 0.848) (Fig. 5). Additionally, no significant difference in BCVA at 1.5 months was detected between prone and nonprone positions (0.41 ± 0.28 LogMAR vs. 0.4 ± 0.31 LogMAR with p value = 0.832).

#### Fluctuations in IOP

Compared with SF6, air had a significantly greater proportion of patients with no increase in IOP (100% in Air vs. 77.59% in SF6) and a significantly lower proportion of patients with an increase in IOP (0% in Air vs. 22.41% in SF6 with p value = 0.015).

Pairwise comparisons revealed significant differences in IOP spikes between the Air + Prone position and the SF6 + Prone position (p value = 0.002) and between the SF6 + Prone position and the SF6 + Non-Prone position (p value = 0.004).

When a comparison of IOP spikes in different positions was done, compared with the nonprone position, the prone position had a comparable distribution of IOP increase: no increase in IOP was detected in 78.18% of patients in the prone position. Whereas the increase in IOP was detected in 21.82% of patients in the nonprone position vs. only 4% of patients in the prone position with a significant p value = 0.054.

Three patients in the nonprone group presented with a shallow anterior chamber and the formation of a PAS with increased IOP at 1 week after surgery. Two patients in the SF6 + nonprone group underwent immediate AC reformation with YAG PIs, and one patient underwent glaucoma drainage device implantation for intractable IOP secondary to PAS formation. The reasons for these complications could be secondary to the anterior push phenomenon, the formation of an iris bombe, a pupillary block and the formation of a peripheral anterior synechaie secondary to gas in the vitreous cavity.

## Discussion

The present study investigated the anatomical and functional outcomes of small- to medium-sized macular hole (MH) closure using different tamponade agents—SF6 and air—in both prone and nonprone positions. The findings provide important insights into the effectiveness of these approaches, contributing to the literature and addressing knowledge gaps regarding the role of positioning and the potential of air tamponade in medium-sized MHs. Most of the literature available for positioning and use of air as tamponade is for small MHs. Most of the studies available in the literature either compare the tamponades or the positioning separately. We compared the variables in the three groups of patients with small- to medium-sized MHs with inverted ILM peeling.

Both air and SF6 combined with prone positioning yielded high rates of anatomical macular hole closure, with the Air + Prone and SF6 + Prone groups achieving greater Type 1 closure rates (87.88% and 90.91%, respectively) than SF6 + Non‒Prone group (60%). These results emphasize the critical role of prone positioning in enhancing tamponade efficacy by maintaining better contact between the MH and the tamponade agent. The significant reduction in the PR layer defect length observed in the SF6 + prone and air + prone groups (*p* = 0.049) further supports the need for prone positioning to promote faster and better anatomical with structural recovery.

In terms of functional recovery, the study did not show any significant difference in visual outcomes. This can be attributed to the significantly better baseline BCVA in the Air + Prone group than in the SF6 + Prone group and SF6 + non prone group (*p* = 0.022), possibly reflecting superior baseline conditions and hence reduced preoperative damage. By 1 month postsurgery, no significant differences in BCVA were observed across groups (*p* = 0.68), suggesting that both air and SF6 tamponades yield comparable visual outcomes over time. This aligns with prior findings by Forsaa et al., who reported favourable visual results for both tamponades in small- to medium-sized macular holes [6]. Near vision assessments also corroborated the equivalence of these treatment modalities at 1 month postoperatively.

The current findings align with and extend existing studies on the efficacy of various tamponade agents, including SF6, air, and C3F8, for MH closure. Previous studies, such as that of Tognetto et al. (2006), highlighted that while silicone oil was not significantly more effective than gas tamponades in terms of anatomical closure, successful visual outcomes were closely tied to anatomical success [7]. Similarly, Hasegawa et al. (2009) and Usui et al. (2013) reported no significant differences in anatomical outcomes between air and SF6, although air offered the advantage of shorter prone positioning durations [8, 9]. A randomized controlled trial (RCT) by Lindtjorn et al. (2022) of 150 patients demonstrated that air tamponade is noninferior to SF6 gas tamponade for macular hole (MH) surgeries of ≤ 400 microns in base-diameter, in prone position. These findings underscore the potential of air tamponade as an alternative to SF6, especially in cases where prolonged positioning is challenging and not physically possible [10]. Eckardt et al. (2008) introduced OCT-based monitoring to minimize prone positioning duration where air was used as tamponade for small-sized macular holes, demonstrating favourable outcomes with reduced patient discomfort in managing prone position. This study demonstrated that prone positioning is crucial for only the first 3 days [11]. Forsaa et al. (2017) further supported the use of air tamponade with nonsupine/prone positioning, in pseudophakic patients with small and medium-sized macular holes [7]. A study by He F et al. also revealed that air can be used as a safe tamponade for the management of MH, with recovery in postoperative photoreceptor layer defects and favourable visual recovery [12]. Despite these advancements, the role of nonprone positioning with SF6 and the broader adoption of air tamponade for medium and large macular holes remain underexplored.

The present study addresses this gap, demonstrating that air tamponade in prone positioning and SF6 in prone positioning achieve similarly high closure rates in medium-sized macular holes. However, the reduced success in nonprone positioning highlights the need for careful patient selection and adherence to positioning protocols, specifically in medium macular holes, for the initial five days postoperatively.

The importance of postoperative positioning was evident in the our study. Compared with prone positioning, even with SF6, nonprone positioning resulted in lower closure rates and a greater proportion of persistent holes (20%). This aligns with studies by Hasegawa et al. and Eckardt et al., who emphasized the biomechanical benefits of prone positioning in achieving optimal tamponade contact with the macular surface and hence greater incidence of macular hole closure [8, 11].

Notably, our study revealed a greater incidence of intraocular pressure (IOP) elevation in the SF6 than in the air (*p* = 0.0002). This further reiterates the importance of vigilant postoperative IOP monitoring in patients receiving SF6, particularly when it is combined with nonprone positioning. The nonprone position predisposes patients to greater incidences of anterior chamber shallowing and iris bombe formation due to the anterior push phenomenon and the formation of the PAS, hence leading to increased IOPs.

However, Eckardt et al. and Usui et al. suggested that shortened prone positioning durations may improve patient compliance without compromising outcomes [9, 11]. Additionally, these studies have shown that MH healing typically occurs within the first three days after surgery, reducing the need for extended face-down or prone positioning. Compliance with a position can significantly impact outcomes, especially in elderly patients. Thus, balancing comfort and efficacy becomes critical in postoperative care strategies.

Ye et al. explored the necessity of postoperative face-down positioning (FDP) and its impact on closure rates, particularly when it was stratified by macular hole size. Their meta-analysis revealed that FDP/prone position significantly improved closure rates for macular holes larger than 400 μm, whereas it appeared unnecessary for smaller holes. These findings support a tailored approach to posturing, potentially reducing the discomfort and complications associated with prolonged prone positioning in patients with small macular holes [13]. This aligns with the findings of our study, where patients in the SF6 tamponade with nonprone positions had more patients with type 2 closure and persistent holes in medium-sized MHs and larger PR layer defects in the nonprone group at postoperative day 10, indicating the importance of the prone position during the initial postoperative days.

The strengths of this study include its randomized controlled design, which minimizes bias and allows robust comparisons between treatment modalities. Standardized surgical techniques and blinded assessments further increase the reliability of the results. Additionally, the inclusion of multiple outcome parameters—anatomical, functional, and structural—provides a comprehensive evaluation of each treatment approach. The operating surgeons had more than 10 years of experience and were familiar with the technique, which added value to the study and negated subjective surgeon performance bias in terms of outcomes.

However, the relatively small sample size of this study may limit its statistical power to detect subtle differences between groups, and the 1.5-month (6 weeks) follow-up period does not capture long-term outcomes, such as delayed closure, late-onset complications, or sustained visual recovery. Future research should focus on larger sample sizes, longer follow-up durations, and the inclusion of quality-of-life measures to better assess the implications of these interventions. If we had used ETDRS visual acuity, we may have found a significant difference in visual acuity among the three groups, justifying our findings of persistent PR defects at the end of the 10th day and final visual outcomes.

This study is unique because it compares three distinct groups, which has not been done in previous research. This study highlights that nonprone positioning leads to poorer outcomes even for small- to medium-sized macular holes. While ellipsoid zone (EZ) or photoreceptor layer defects were significantly more common in patients in the nonprone group, the defects were less severe in the prone position group. This underscores the importance of prone positioning, even for a short duration of 3–5 days, regardless of the tamponade agent used.

In conclusion, this study highlights the critical importance of postoperative positioning and tamponade agent selection in achieving optimal outcomes for small to medium macular holes (MHs). While air tamponade combined with prone positioning has a comparable anatomical and functional success rate to SF6 with prone positioning, it offers the added benefit of reducing intraocular pressure (IOP)-related complications and early visual recovery as air diffuses faster, making it a viable alternative. In contrast, nonprone positioning even with long-acting gases such as SF6 is linked to lower closure rates and higher incidences of persistent holes as well as larger ellipsoid zone/PR layer defects in the postoperative period, emphasizing the necessity of prone positioning even for small MHs. These findings provide valuable insights for refining surgical strategies aimed at enhancing patient outcomes and minimizing complications. Further research with larger sample sizes and extended follow-up periods is needed to confirm these results and explore innovative, patient-centered approaches to postoperative care. Impact of earlier resolution of air and/or non prone positioning along with earlier visual rehabilitation.

## Data Availability

No datasets were generated or analysed during the current study.
